# A Combined Water Extract of Frankincense and Myrrh Alleviates Neuropathic Pain in Mice via Modulation of TRPV1

**DOI:** 10.1155/2017/3710821

**Published:** 2017-06-27

**Authors:** Danyou Hu, Changming Wang, Fengxian Li, Shulan Su, Niuniu Yang, Yan Yang, Chan Zhu, Hao Shi, Lei Yu, Xiao Geng, Leying Gu, Xiaolin Yuan, Zhongli Wang, Guang Yu, Zongxiang Tang

**Affiliations:** ^1^Key Laboratory for Chinese Medicine of Prevention and Treatment in Neurological Diseases, Nanjing University of Chinese Medicine, 138 Xianlin Rd, Nanjing, 210023 Jiangsu, China; ^2^School of Medicine and Life Sciences, Nanjing University of Chinese Medicine, 138 Xianlin Rd, Nanjing, 210023 Jiangsu, China; ^3^Department of Anesthesiology, Zhujiang Hospital of Southern Medical University, 253 Gongye Rd, Guangzhou, 510282 Guangdong, China; ^4^Jiangsu Key Laboratory for High Technology Research of TCM Formulae, Nanjing University of Chinese Medicine, 138 Xianlin Rd, Nanjing, 210023 Jiangsu, China

## Abstract

Frankincense and myrrh are widely used in clinics as a pair of herbs to obtain a synergistic effect for relieving pain. To illuminate the analgesia mechanism of frankincense and myrrh, we assessed its effect in a neuropathic pain mouse model. Transient receptor potential vanilloid 1 (TRPV1) plays a crucial role in neuropathic pain and influences the plasticity of neuronal connectivity. We hypothesized that the water extraction of frankincense and myrrh (WFM) exerted its analgesia effect by modulating the neuronal function of TRPV1. In our study, WFM was verified by UHPLC-TQ/MS assay. In vivo study showed that nociceptive response in mouse by heat and capsaicin induced were relieved by WFM treatment. Furthermore, thermal hypersensitivity and mechanical allodynia were also alleviated by WFM treatment in a chronic constriction injury (CCI) mouse model. CCI resulted in increased TRPV1 expression at both the mRNA and protein levels in predominantly small-to-medium neurons. However, after WFM treatment, TRPV1 expression was reverted in real-time PCR, Western blot, and immunofluorescence experiments. Calcium response to capsaicin was also decreased in cultured DRG neurons from CCI model mouse after WFM treatment. In conclusion, WFM alleviated CCI-induced mechanical allodynia and thermal hypersensitivity via modulating TRPV1.

## 1. Introduction

The main characteristics of neuropathic pain are allodynia, hyperalgesia, and persistent pain [1, 2], which changes the quality of life for millions of people worldwide. Massive studies have been designed to disclose the precise mechanisms [3, 4]. However, the randomized clinical trial drugs have shown that the analgesic effect is less than that of patients treated with conventional drugs [5]. This prompts us to find new strategies for the affliction. There is growing interest in herbal remedies. Clinical data have shown promising effects of multiple herbs including frankincense and myrrh in pain relief [6].

Frankincense is the dried gum resin of *Boswellia carterii,* one of 43 species in the genus *Boswellia* of the *family Burseraceae*. It has been commonly used to alleviate pain in different diseases [7, 8]. In vitro studies have shown that boswellic acids, which are isolated from frankincense, have the potential to regulate immune function [9]. Myrrh is an aromatic gum resin, which is the plant stem resinous exudate of *Commiphora myrrha* (Nees) Engl. (*Burseraceae*) and other different species of the *Commiphora* family. Myrrh is widely used in clinics in India, China, Rome, and Greece to treat painful diseases such as ache and dysmenorrhea [10]. Pharmacological studies have shown that myrrh has multiple activities (effects), including anti-inflammatory and antimicrobial [11, 12]. However, the mechanism is not fully understood for frankincense and myrrh, which are used as a pair of herbs to relieve pain sensation. Although several elements are thought to be the key mechanisms—including reactive oxygen species and inflammatory cytokines for their antinociceptive effect, the precise molecular mechanisms are still obscure [13].

The transient receptor potential vanilloid 1 (TRPV1) is a nonselective cation channel involved in the detection and transduction of nociceptive stimulus [14]. Upregulation of TRPV1 transcription can be induced by inflammation and nerve damage. Modulating of TRPV1 activity is considered an effective strategy in treating inflammatory and neuropathic pain conditions [15, 16]. Hence, TRPV1 has become a promising target for screening analgesics via either blocking the function of the receptor or eliminating the nociceptor by utilizing a high dose of agonists [17–19].

In China, formula is commonly used in pain treatment. The main herb pair is recognized to be the most important part of the formula. Frankincense and myrrh as an herb pair has shown promising effects in pain relief. It is possible that they might have the potential of alleviating neuropathic pain by modulating TRPV1. However, there is almost no literature report on this pair of herb to relieve neuropathic pain by regulating TRPV1. Here, we obtained WFM from frankincense and myrrh in boiled water and verified some effective components by UHPLC-TQ/MS assay. A CCI mouse model was then conducted to elucidate the modulating effect of WFM on TRPV1, which achieved the pain relief effect. Furthermore, we checked the inhibition effect of WFM on the expression, sensitivity of TRPV1.

## 2. Materials and Methods

### 2.1. WFM Extraction and UHPLC-TQ/MS Assay

The frankincense and myrrh were purchased from the Jiangsu Traditional Chinese Medical Hospital (Nanjing, China), identified and authenticated by Dr. Shulan Su in the College of Pharmacy, Nanjing University of Chinese Medicine. Chemical standards including *β*-boswellic acid, 3*α*-acetoxy-tirucall-7,24-dien-21-oic acid, 3-aectyl-11-keto-*β*-boswellic acid, 3-keto-tirucall-8,24-dien-21-oic acid, and abietic acid were separated and identified in previous studies. The dry herb of frankincense (100 g) and myrrh (100 g) were extracted from boiling water (1600 ml) twice and filtered through gauze. Filtrates were then evaporated by rotary evaporation under vacuum at 55°C. Finally, a semi dry mass of about 50 g was obtained and used in the experiment.

The UHPLC-TQ/MS method was applied for determining the components of WFM as follows: Chromatographic analysis was performed on a Waters Acquity UHPLC system (Waters, Corp., Milford, MA, USA), consisting of a binary pump solvent management system, an online degasser, and an autosampler. An Acquity™ UPLC BEH C_18_ column (2.1 mm × 50 mm, 1.7 *μ*m) was employed, and the column temperature was maintained at 30°C. The mobile phase was composed of A (acetonitrile) and B (0.1% formic acid) using a gradient elution of 10% A at 0-1 min, 5% A at 1–9 min, and 10% A at 9-10 min with a flow rate set at 0.4 ml·min^−1^. The sample injection volume was 2 *μ*l. The ESI source was set in both positive and negative ionization mode. The scanning mode was set multiple reaction monitoring (MRM) mode, and the range of selected monitor ion was m/z 100–m/z 1000. The TQ mass spectrometer was operated with a capillary voltage of 3.5 kV, a sampling cone voltage of 35 V, and a capillary temperature of 275°C. The Helium gas flow was at 35 arb, and the auxiliary gas flow rate was at 15 arb. All of the data acquisition and analyses of data were controlled by Waters MassLynx v4.1 software. The cone voltage and collision energy optimized for each analyte and selected values are given in Table 1.

### 2.2. Animals

All experiments were performed under protocols approved by the Animal Care and Use Committee of the Nanjing University of Chinese Medicine. All mice used in the behavioral tests were 8- to 10-week old males in a C57Bl/6 background (WT mice). Tested animals were housed, and behavior experiments were performed in a controlled environment of 20–24°C, humidity of 45–65%, and with a 12-hour day/night cycle.

### 2.3. CCI Model and Treatment

**Figure 1:**
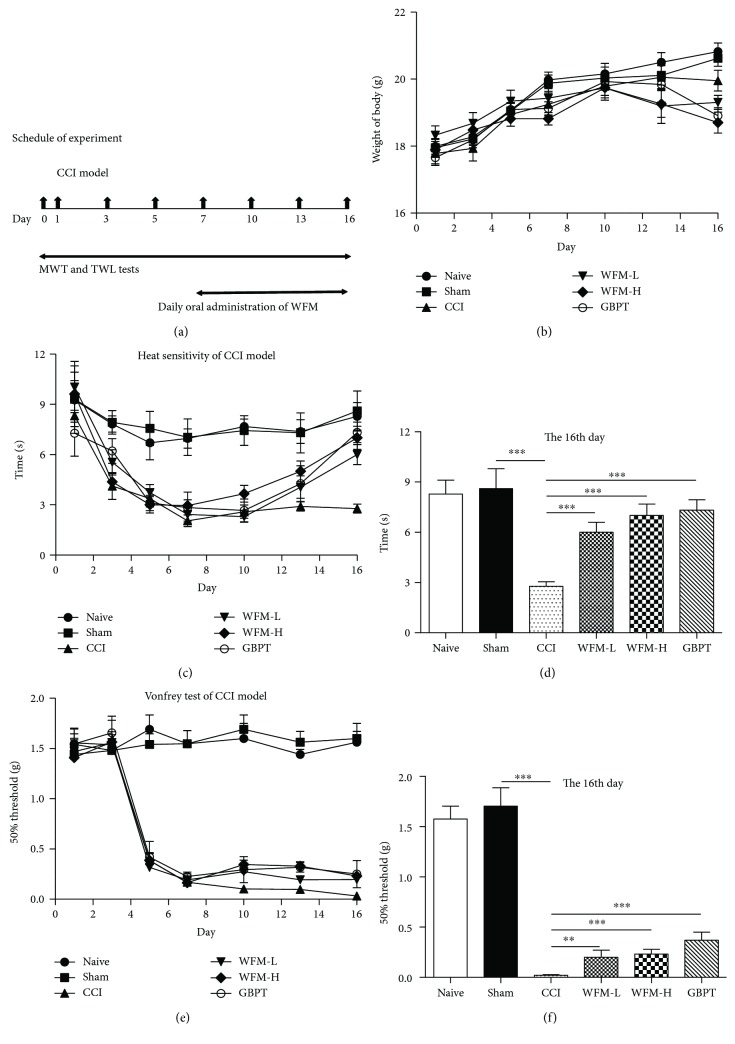
Effects of WFM on chronic constriction injury (CCI) of sciatic nerve treated mouse. (a) Schedule of CCI model and WFM treatment. (b) There was no difference in the body weight after vehicle or drug treatment among six groups. (c, d) Effects of WFM in the thermal withdrawal latency (TWL) was recorded (*n* = 12). (e, f) Effects of WFM in the mechanical withdrawal threshold (MWT) was recorded (*n* = 12). ^∗^*p* < 0.05, ^∗∗^*p* < 0.01, ^∗∗∗^*p* < 0.001.

Unilateral CCI surgery was performed in mice under chloral hydrate anesthesia (50 mg/kg, i.p.). Briefly, after skin preparation and iodine complex disinfection, the skin was incised and muscles were bluntly separated. The right sciatic nerve was found at the mid-thigh level. Then two ligations with gut were performed loosely on the nerve, each spaced 1 mm apart. Mice in the sham group received the same procedure without sciatic nerve hypodesmus. Seventy two male mice were randomly divided into six groups (*n* = 12 per group) based on the treatment, naïve + vehicle (distilled water), sham + vehicle, CCI + vehicle, CCI + WFM-L (WFM 1.5 g/kg/day), CCI + WFM-H (WFM 7.5 g/kg/day), and CCI + GBPT (Gabapentin) as a positive control treatment (delivered at 0.2 g/kg/day). All mice received vehicle or drug treatment from 7th day to 16th day (Figure 1(a)). The same volume of drugs or vehicle was administrated blindly by intragastric gavage by the same person. No mice or data points were excluded.

### 2.4. Behavioural Assay

Animals were acclimated to the testing environment for 10 minutes before the initiation of behavior tests. Animal behavior was analyzed by investigators who were blind to the grouping and treatment. The tail-flick experiments were carried out as previously reported in the 50°C water bath [20]. Mice were gently restrained in a towel and handheld. Approximately 1 cm of the tip of the tail was submerged in a hot water bath maintained at 50°C, and the latency to withdraw the tail was measured. Capsaicin (3 *μ*g/mouse) was injected into the dorsal surface of the right hind paw 3 hours after WFM administration. Licking and biting behavior induced by subcutaneously injected capsaicin were observed for 15 minutes. Mechanical withdrawal threshold (MWT) and thermal withdrawal latency (TWL) were recorded at the 1st, 3rd, 5th, 7th, 10th, 13th, and 16th day. Mice were treated with WFM (once/day) 7 days after unilateral CCI, when all injured mice have developed hindpaw mechanical allodynia and thermal hypersensitivity on the injured side.

### 2.5. DRG Neuron Culture and Calcium Imaging

DRGs (L4-5 and S1–3) were dissected from different groups of tested mice and collected in DH10 medium on ice (90% DMEM/F-12, 10% FBS, 100 U/ml penicillin, 100 mg/ml streptomycin, Gibco). Dissected DRGs were then digested for 25 minutes at 37°C in a protease solution (5 mg/ml dispase, 1 mg/ml collagenase type I in HBSS without Ca^2+^ and Mg^2+^, and Gibco) before being triturated to free neurons and pelleted by centrifugation. Pelleted neurons were then resuspended in DH10 medium supplemented with NGF (20 ng/ml) and GDNF (25 ng/ml) and plated onto glass coverslips coated with poly-D-lysine (0.5 mg/ml, Sigma) and laminin (10 mg/ml, Sigma). Neurons were cultured in an incubator (95% O_2_ and 5% CO_2_) for 24 hours before they were used for calcium imaging [21]. Neurons were loaded with Fura 2-acetomethoxy ester (Molecular Probes) for 30 min at room temperature. After washing and recovery for 5 min, cells were imaged at 340 and 380 nm excitation to detect the intracellular-free calcium. Cells were considered responding if their fluorescence ratio is greater than or equal to 0.5 (fluorescence ratio = ΔF/F0, ΔF means maximal value of fluorescence-baseline value of fluorescence, F0 means baseline value of fluorescence). Calcium imaging assays were performed with an experimenter blind to the grouping [22]. Each test was done three times.

### 2.6. RNA Extraction and Real-Time PCR

Total RNA from freshly dissected DRGs (L4–5 and S1–3) was isolated and purified using a TRIzol/chloroform (Life Technologies, Carlsbad, California, USA) and an isopropanol precipitation procedure in accordance with the manufacture's protocols. cDNA was compiled using the Transcript First Strand cDNA Synthesis Kit (Roche, Basel, Switzerland). Real-time PCR was performed using LightCycler 480 SYBR Green 1 Master Mix (Roche, Basel, Switzerland) and a LightCycler 480 2 Real-Time PCR instrument (Roche, Basel, Switzerland). Briefly, 1 *μ*l of cDNA from each sample was used for reaction. Primers (forward primer: ATCATCAACGAGGACCCAGG, reverse primer: TGCTATGCCTATCTCGAGTGC) were used to amplify TRPV1 expression. Calibrations and normalizations were done using the 2^-△△CT^ method, where △△CT = (CT (target gene) − CT (reference gene)) − (CT (calibrator) − CT (reference gene)). GAPDH was used as the reference gene for real-time PCR experiments (forward primer: TGGATTTGGACGCATTGGTC, reverse primer: TTTGCACTGGTACGTGTTGAT). After real-time quantification, amplification products were analyzed by electrophoresis on 1.5% agarose gel for band size consistency.

### 2.7. Protein Extraction and Western Blotting

Total protein from freshly dissected DRGs (L4-5 and S1–3) was isolated and purified using a TRIzol/chloroform (Life Technologies, Carlsbad, California, USA) and an isopropanol precipitation procedure after RNA extraction in accordance with the manufacture's protocols. The protein concentration was determined by BCA assay. *β*-actin was selected as an internal control. Polyclonal antibody of TRPV1 (Neuromics, USA) was used at 1/500 dilution, and a monoclonal antibody of *β*-actin (Santa Cruz Biotechnology, Dallas, TX) was used at a 1/1000 dilution. Equal quantities of protein (60 *μ*g per lane) were resolved on 12% SDS-polyacrylamide gels. Western blotting was performed as detailed previously [23]. The intensity of the signals was used to estimate the relative concentration of TRPV1 protein in the DRG extracts.

### 2.8. Immunohistochemical Staining

Mice were anesthetized with 1% sodium pentobarbital (50 mg/kg, i.p.) and transcardially perfused with 0.1 M phosphate-buffered saline (PBS, pH 7.4, 4°C) followed by 4% paraformaldehyde in PBS (pH 7.4, 4°C). DRGs (L4-5) were dissected from perfusion mice and postfixed in 4% paraformaldehyde in PBS for 30 minutes and cryoprotected in 30% sucrose at 4°C for 24 hours. DRGs were then embedded in an optimum cutting temperature compound (OCT, Leica, Wetalar, Germany) and rapidly frozen at −20°C (CM1950, Leica). Cryoembedded tissues were then cut into 20 *μ*m thick slices using a sliding microtome (CM1950, Leica).

Sectioned DRGs were incubated in blocking solution (10% fetal bovine serum in PBS containing 0.1% Triton X-100) for 30 min at room temperature, followed by anti-TRPV1 (1 : 500; Neuromics, USA) at 4°C overnight. Afterwards, tissue sections were washed with 0.1% PBST and incubated in secondary antibody (1 : 200; Beyotime Biotechnology, China) at room temperature for 2 hours in the dark. Sections were then washed with 0.1% PBS and mounted with glycerol. All imaging was performed with an Olympus fluorescence microscope (BX51, Olympus Japan). Three mice from each group were analyzed. Quantitative analysis of immune response was consistent with previous studies [24].

### 2.9. Data Analysis

All data are presented as mean ± SEM. Statistical comparisons are performed using 2-tailed Student's *t*-tests. The difference is considered statistically significant at *p* < 0.05.

## 3. Results

### 3.1. UHPLC-TQ/MS Identified and Determined WFM

**Figure 2:**
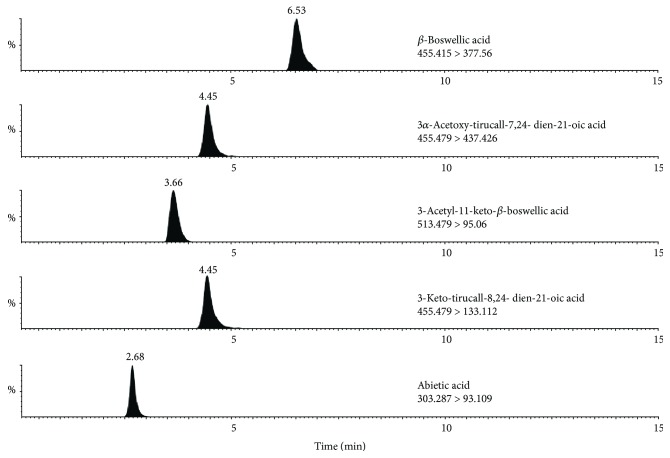
The UHPLC-TQ/MS method was adopted to qualitify the main bioactive components of WFM. By comparing the characterization of tR, λmax, and m/z with standard compounds, *β*-boswellic acid, 3*α*-acetoxy-tirucall-7,24-dien-21-oic acid, 3-aectyl-11-keto-*β*-boswellic acid, 3-keto-tirucall-8,24-dien-21-oic acid, and abietic acid were used as the marker to identify the WFM.

Five main compounds of *β*-boswellic acid, 3*α*-acetoxy-tirucall-7,24-dien-21-oic acid, 3-acetyl-11-keto-*β*-boswellic acid, 3-keto-tirucall-8,24-dien-21-oic acid, and abietic acid in the WFM were identified and determined by the UHPLC-TQ/MS method (Figure 2). They were by comparing the characterization of t_R_, λ_max_, and m/z with standard compounds. The percentage composition of these five compounds in the WFM extraction were 1.07%, 2.02%, 1.65%, 0.92%, and 0.28%, respectively.

### 3.2. WFM Alleviated Nociceptive Behavior in Mice

**Figure 3:**
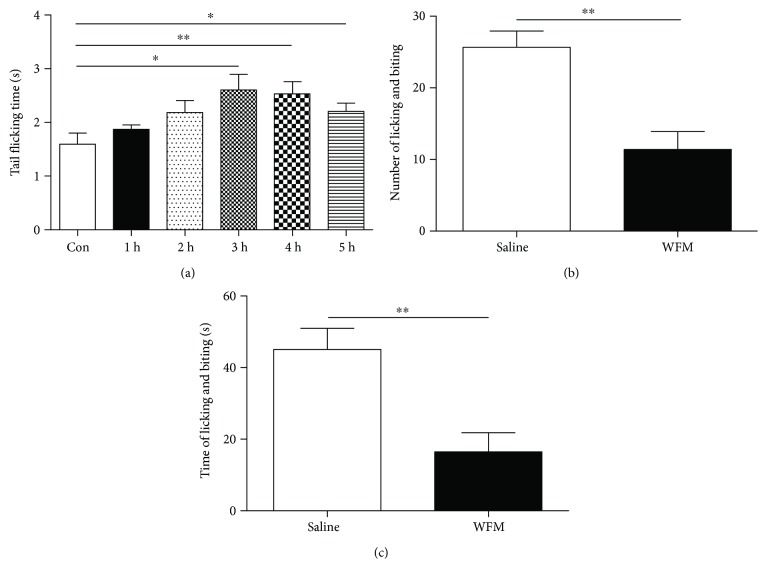
Antinociceptive effects of WFM in the tail-flick and capsaicin injection assay. (a) Antinociceptive effects in the tail-flick assay. WT mice were used in this assay. The time course of the tail-flick latencies was observed after WFM treatment (*n* = 8). (b, c) Effects of WFM in the capsaicin assay. WT mice were used in this assay (*n* = 8). (b) Number of licking and biting. (c) Time of licking and biting. Two-way ANOVA revealed significant drug effect. ^∗^*p* < 0.05, ^∗∗^*p* < 0.01, ^∗∗∗^*p* < 0.001.

Thermal pain is a typical form of pain in daily life. The high temperature water bath of 50°C induced tail-flicking behavior in a short time in mice (1.6 ± 0.21 s) without WFM treatment in our experiment. However, WFM by intragastric gavage (1.5 g/kg) significantly increased the delay tail-flicking behavior in a dose-dependent manner after 3 hours (2.6 ± 0.29 s) and 4 hours (2.5 ± 0.23 s); the data were shown in Figure 3(a) (*P* < 0.05 and *P* < 0.01, respectively). To extend our understanding of its antinociceptive efficacy, we tested WFM in the capsaicin assay. We were surprised to find that WFM significantly alleviated capsaicin-induced licking and biting response by intragastic gavage. The bouts of licking and biting were decreased from 26 ± 2.3 s to 11 ± 2.5 s, while the time spent on licking and biting was also significantly decreased from 45 ± 6.0 s to 17 ± 5.4 s during a 5 min period (Figures 3(b) and 3(c), *P* < 0.01).

### 3.3. WFM Alleviated Nociceptive Behavior in a CCI Mouse Model

Nerve ligation is commonly used as an animal model of neuropathic pain, which induces mechanical allodynia and thermal hypersensitivity [25]. The mechanical withdrawal threshold (MWT) and thermal withdrawal latency (TWL) of each experimental animal were recorded as baseline before CCI model (day 0). Then MWT and TWL were measured once every two days until the entire experiment was completed (a total of 16 days). From the 7th day after CCI model, mice were treated with WFM once a day. The experimental process of animal behavior was shown in Figure 2. There was no difference in the body weight among six group experiments (Figure 1(b)). Thermal hypersensitivity and mechanical allodynia appeared a significant difference from 3rd day to 5th day in CCI model (Figures 1(c) and 1(e)). However, after treatment with WFM on the 7th day, the temperature and mechanical pain behaviors of mice were improved gradually. The effect of WFM on pain relief has become apparent on the 16th day, and the roles of this relief will remain if WFM continues to be administered. The analgesic of WFM was dose-dependent, and the high concentration of WFM had strong effect. The role of WFM is almost the same as that of gabapentin (GBPT) which is a common clinical analgesic drug (Figures 1(d) and 1(f)). Because there were no difference between naïve and sham group in MWT and TWL tests (Figures 1(c) and 1(e)), we chose the sham group as a control in other experiments including q-PCR, Western blot, immunohistochemical staining, and calcium imaging.

### 3.4. WFM Attenuated TRPV1 Expression in CCI Model

**Figure 4:**
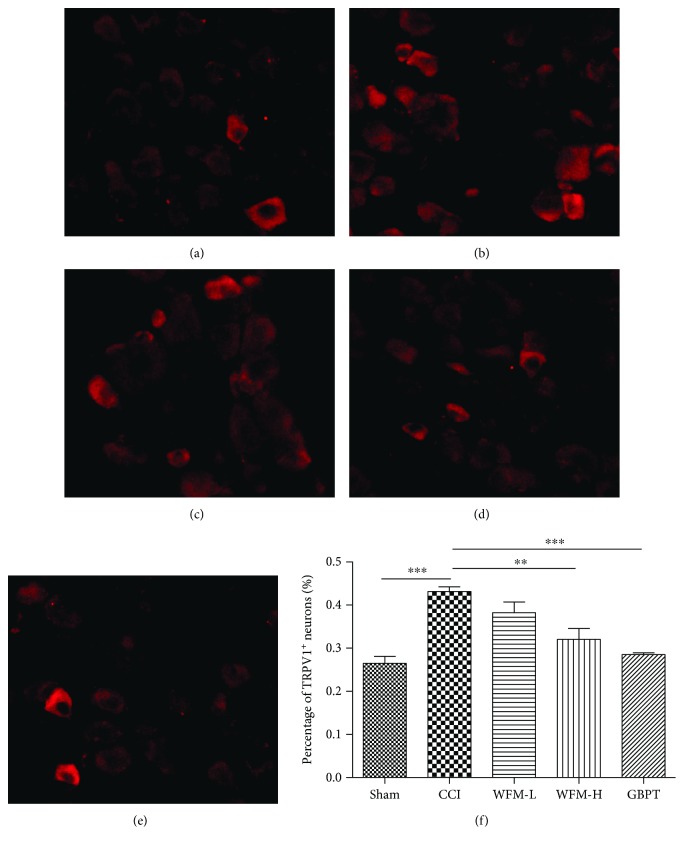
TRPV1^+^ neuron is increased in CCI model and can be restored by WFM treatment. Histochemistry staining of DRG from CCI model and WFM treatment groups. (a, b) The proportion of TRPV1^+^ population is increased in CCI model. (c, d) The fraction TRPV1^+^ neuron in DRG is significantly decreased after WFM treatment. (e) The fraction TRPV1^+^ neuron in DRG is significantly decreased after GBPT treatment. (f) Data are presented as mean ± SEM, ^∗^*p* < 0.05, ^∗∗^*p* < 0.01, ^∗∗∗^*p* < 0.001. Scale bar: 50 *μ*m.

**Figure 5:**
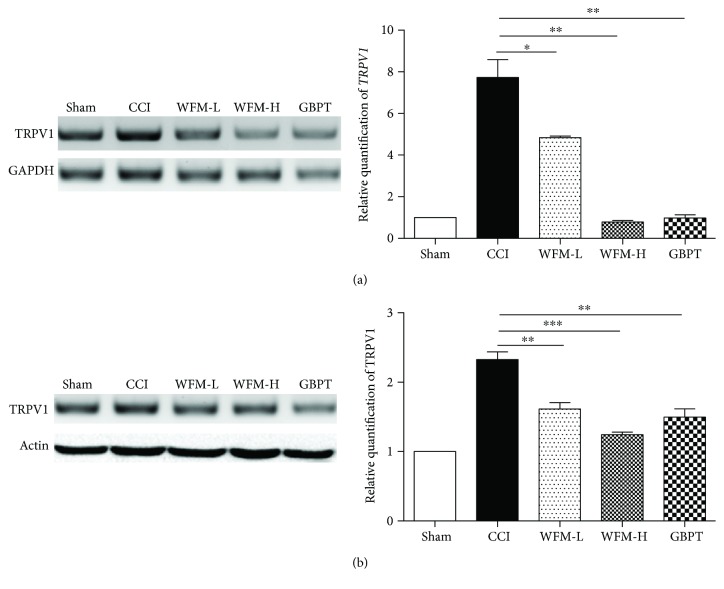
TRPV1 expression is significantly decreased in the DRG after WFM treatment. (a) Real-time PCR results indicate that *TRPV1* expression is decreased after WFM treatment. (b) Western blot results confirm that TRPV1 expression is decreased after WFM treatment. ^∗^*p* < 0.05, ^∗∗^*p* < 0.01, ^∗∗∗^*p* < 0.001.

To reveal the cellular mechanism of WFM relieving nociceptive behavior in CCI model, the TRPV1 expression level was examined in L4-5 and S1–3 DRGs. The percentage of TRPV1 immunoreactive neurons significantly increased from 26.4 ± 1.63% in the sham group to 43.1 ± 1.09% in the CCI group and then attenuated from 43.1 ± 1.09% to 38.3 ± 2.45% in the low-dose group (WFM-L) and 32.1 ± 2.50% in the high-dose group (WFM-H) after WFM treatment (*P* < 0.001). The inhibitory effect of WFM was weaker than that of GBPT (28.5 ± 4.21%) in our experiment (Figure 4). These results combined with other experiments including real-time PCR and Western blot assays confirmed that the expression of TRPV1 in the DRGs was significantly increased in CCI model and was inhibited by WFM. GBPT as a positive control also showed a significant inhibitory effect (Figure 5).

### 3.5. WFM Attenuated the Response of DRG Neurons to Capsaicin in CCI Model

**Figure 6:**
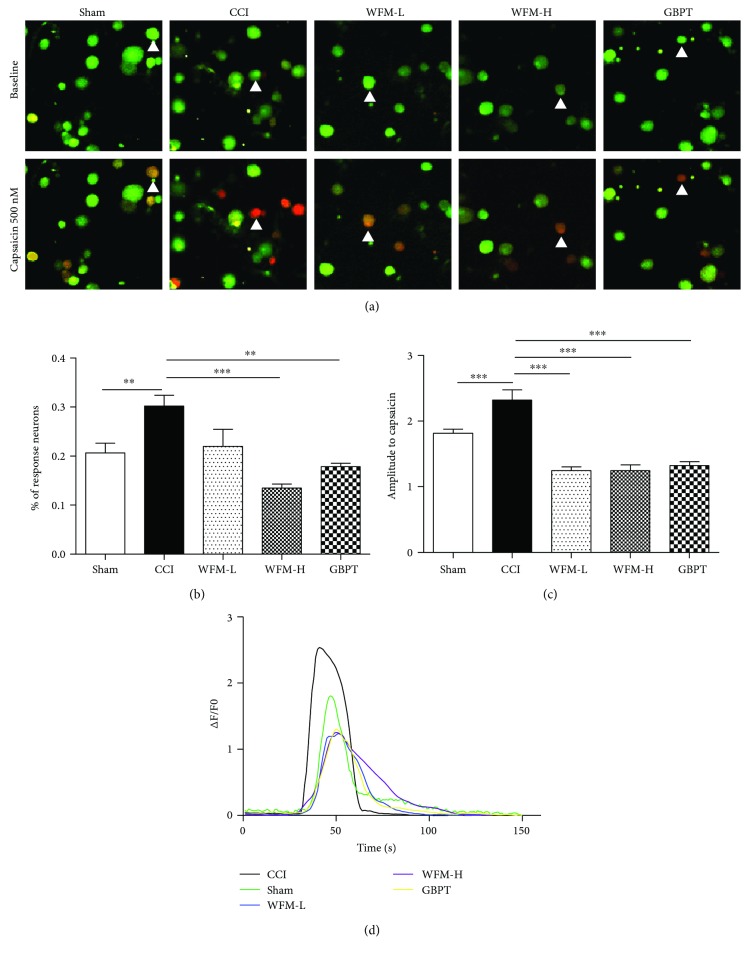
WFM attenuated capsaicin-induced response in CCI-treated sensory neurons. (a) Representative calcium images in cultured DRG neurons from CCI contralateral and ipsilateral. WFM and GBPT could significantly inhibit the responses of DRG neurons from CCI ipsilateral to 500 nM capsaicin. (b) The percentage of reactive neurons to 500 nM capsaicin stimulus. The percentage of DRG neuron response to 500 nM capsaicin stimulus was significantly higher in the ipsilateral (CCI) than in the contralateral (control). WFM and GBPT could significantly reduce the response percentage of DRG neurons to the same concentration of capsaicin stimulus. (c) The amplitude of the response neurons to 500 nM capsaicin stimulus. The amplitude of DRG neurons response to 500 nM capsaicin stimulus was significantly larger in the ipsilateral (CCI) than in the contralateral (control). Both WFM (high dose and low dose) and GBPT could effectively inhibit capsaicin-induced response. (d) Calcium imaging response curves of DRG neurons to different stimulus. White arrows indicate these active neurons. ^∗^*p* < 0.05, ^∗∗^*p* < 0.01, ^∗∗∗^*p* < 0.001.

We further studied the sensitivity of DRG neurons from CCI model mice to capsaicin. The DRG neurons from CCI ipsilateral and contralateral on the same segment were cultured in different culture dishes. Capsaicin was used to activate these DRG neurons. The results indicated that both the ratio of response neurons (from 0.21 ± 0.020 to 0.30 ± 0.022, *P* < 0.01) and the amplitude of response neurons (from 1.8 ± 0.06 to 2.3 ± 0.15, *P* < 0.001) significantly increased. However, before capsaicin stimulus with WFM treatment, both the ratio and the amplitude of response DRG neurons significantly reduced. Low-dose WFM attenuated the ratio of DRG neurons to capsaicin response from 0.30 ± 0.022 to 0.22 ± 0.021(*P* < 0.001) and the amplitude from 2.3 ± 0.15 to 1.3 ± 0.05 (*P* < 0.001). High-dose WFM attenuated the ratio of DRG neurons to capsaicin response from 0.30 ± 0.022 to 0.13 ± 0.008 (*P* < 0.001) and the amplitude of response neurons from 2.3 ± 0.15 to 1.2 ± 0.09 (*P* < 0.001). The attenuated effect of WFM was almost the same as that of GBPT in our experiment (Figure 6).

## 4. Discussion

Nociceptors conduct noxious information from sensory nerve endings to the central nervous system; hence, inhibition of the peripheral nociceptor activity can effectively attenuate pain sensation. It is well-known that TRPV1 acts as an integrator of painful stimuli, which has become a promising target for screening analgesics [26]. Either blocking the function of the receptor or utilizing the lasting loss of function of the receptor is believed to be the effective treatment, especially under pathological pain conditions [27]. However, drugs based on pharmacologically synthesis of TRPV1 antagonists and agonists have failed to achieve the ideal therapeutic effects [28]. Hyperthermia and impaired noxious heat sensation were discovered to be the main obstacle in preclinical studies and clinical trials [29].

TRPV1 enhancement is thought to be the critical element during inflammatory and neuropathic pain, so therapeutic strategies have focused on the modulating effects of TRPV1 with antagonists [30]. Studies have indicated that downregulated TRPV1 expression might attenuate neuropathic pain [31, 32]; hence, new strategies have focused on the regulation or modulation of TRPV1 functions to produce better outcomes and fewer side effects. In the history of drug developmental, the discovery of many new drugs comes from the bioactive compounds of herbal medicines, which are natural or long-tested by traditional usage. Among herbal medicines, frankincense and myrrh have been widely used as clinical treatment for various pain diseases because of their effects, so frankincense and myrrh should be a good candidate for relieving neuropathic pain. Our experiment showed that WFM effectively relieved heat and capsaicin-induced pain in normal conditions as well as attenuated heat hypersensitivity and mechanical allodynia in a CCI mouse model. Our study also showed that the analgesic effect of a high dose of WFM was similar to GBPT, which is one of the classical antipain drugs [33]. Furthermore, this antinociceptive effect of WFM is related to the inhibition of TRPV1 expression and activation. In our study, the inhibition effect of WFM on behavior and calcium response was dramatic, which was directly related to the downregulation of TRPV1 expression and sensitivity. Although NGF can enhance the activity of TRPV1, both the ratio and the amplitude of the response to capsaicin were significantly reduced in cultured DRG neurons after WFM treatment in our experiment. This perhaps due to CCI model led to greater effect in the activity of TRPV1 than NGF. Actually, neurons cultured with NGF also showed different activity to 50°C stimulus. Hence, this indicated that WFM is a potential analgesic which is targeted to TRPV1.

Nevertheless, the antinociceptive effect of WFM is from the multiple elements in the extraction; however, whether the monomers within the WFM have the same effect on TRPV1 modulation is still unknown. Further studies should focus on the characteristics of WFM on human antipain effects.

## 5. Conclusion

In summary, our results showed the efficacy of WFM in relieving CCI-induced hyperalgesia in a mouse model, which shed light on effective and novel therapies for pain management via downregulating TRPV1. Further observations should be conducted to reveal the antipain characteristics of WFM in further studies.

## Figures and Tables

**Table 1:** The cone voltage and collision energy optimized for each analyte and selected values.

Analytes	Ionization mode	MRM transitions (precursor-product)	Cone voltage (V)	Collision energy (eV)
*β*-Boswellic acid	ES^−^	455.415 → 377.356	44	30
3*α*-Acetoxy-tirucall-7,24- dien-21-oic acid	ES^+^	455.479 → 437.426	14	8
3-Acetyl-11-keto-*β*-boswellic acid	ES^+^	513.479 → 95.06	40	42
3-Keto-tirucall-8,24-dien-21-oic acid	ES^+^	455.479 → 133.112	14	36
Abietic acid	ES^+^	303.287 → 93.109	16	30

## Abbreviations

CCI: Chronic constriction injury
DRG: Dorsal root ganglia
MWT: Mechanical withdrawal threshold
TRPV1: The transient receptor potential vanilloid 1
TWL: Thermal withdrawal latency
WFM: Water extract of frankincense and myrrh.
